# Supplementary material for: Atomistic QM/Classical Modeling of Surface-Enhanced Infrared Absorption
Source: J Phys Chem C Nanomater Interfaces. 2026 Jan 22;130(5):1919–30. doi: 10.1021/acs.jpcc.5c07549 (PMC12884528; doi:10.1021/acs.jpcc.5c07549)
Supplement: Supplementary file 1 [file jp5c07549_si_001.pdf]

Supporting Information for: Atomistic QM/Classical Modeling of Surface-Enhanced Infrared Absorption

Sveva Sodomaco,[†] Piero Lafiosca,[†] Tommaso Giovannini,[*,‡,¶] and Chiara Cappelli[*,†,§]

†*Scuola Normale Superiore, Classe di Scienze, Piazza dei Cavalieri 7, 56126, Pisa, Italy*

‡*Department of Physics and INFN, University of Rome Tor Vergata, Via della Ricerca Scientifica 1, 00133, Rome, Italy*

¶*Consorzio Interuniversitario Nazionale per la Scienza e Tecnologia dei Materiali (INSTM), UdR Roma Tor Vergata, Via della Ricerca Scientifica 1, 00133, Rome, Italy*

§*Consorzio Interuniversitario Nazionale per la Scienza e Tecnologia dei Materiali (INSTM), UdR Pisa-SNS, Piazza dei Cavalieri 7, 56126 Pisa, Italy.*

E-mail: tommaso.giovannini@uniroma2.it; chiara.cappelli@sns.it

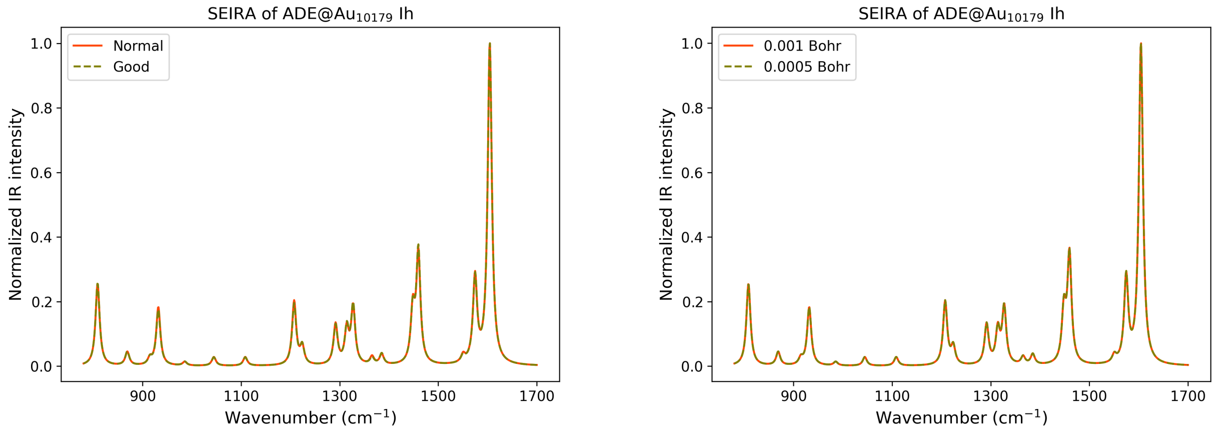

Figure S1: QM/ωFQFμ SEIRA spectra of Adenine on Au_{10179} Ih (in v_p morphology) computed by exploiting DFT integration grids of increasing accuracy (from "NumericalQuality Normal" to "Numerical Quality Good"; left) and by varying the differentiation step for numerical differentiation (from 0.0005 to 0.001 Bohr; right).

a)

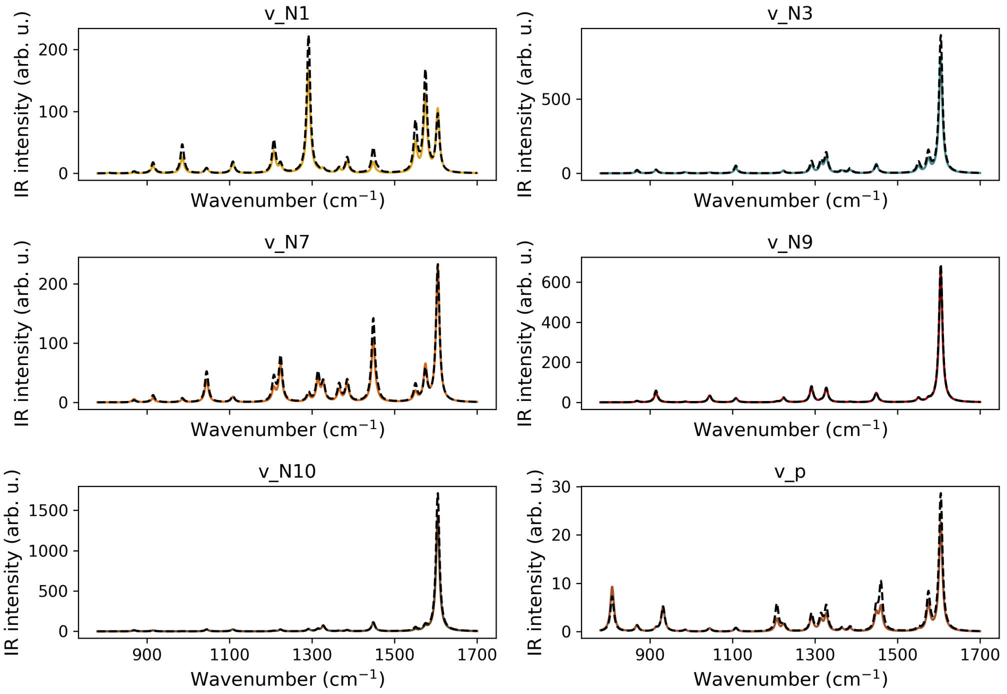

b)

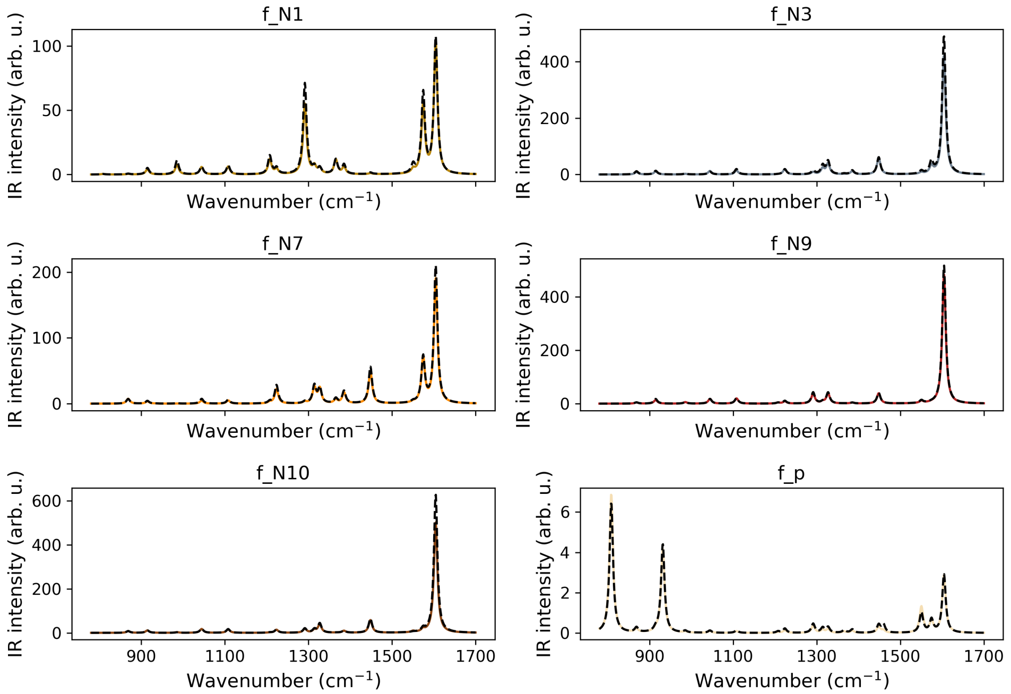

Figure S2: QM/ωFQFμ SEIRA spectra of ADE for the six configurations on the vertex (a) and face (b) of Au_{10179} Ih computed by including (black dashed lines) or not (colored solid lines) the FQFμ contribution to the ground state Hamiltonian.

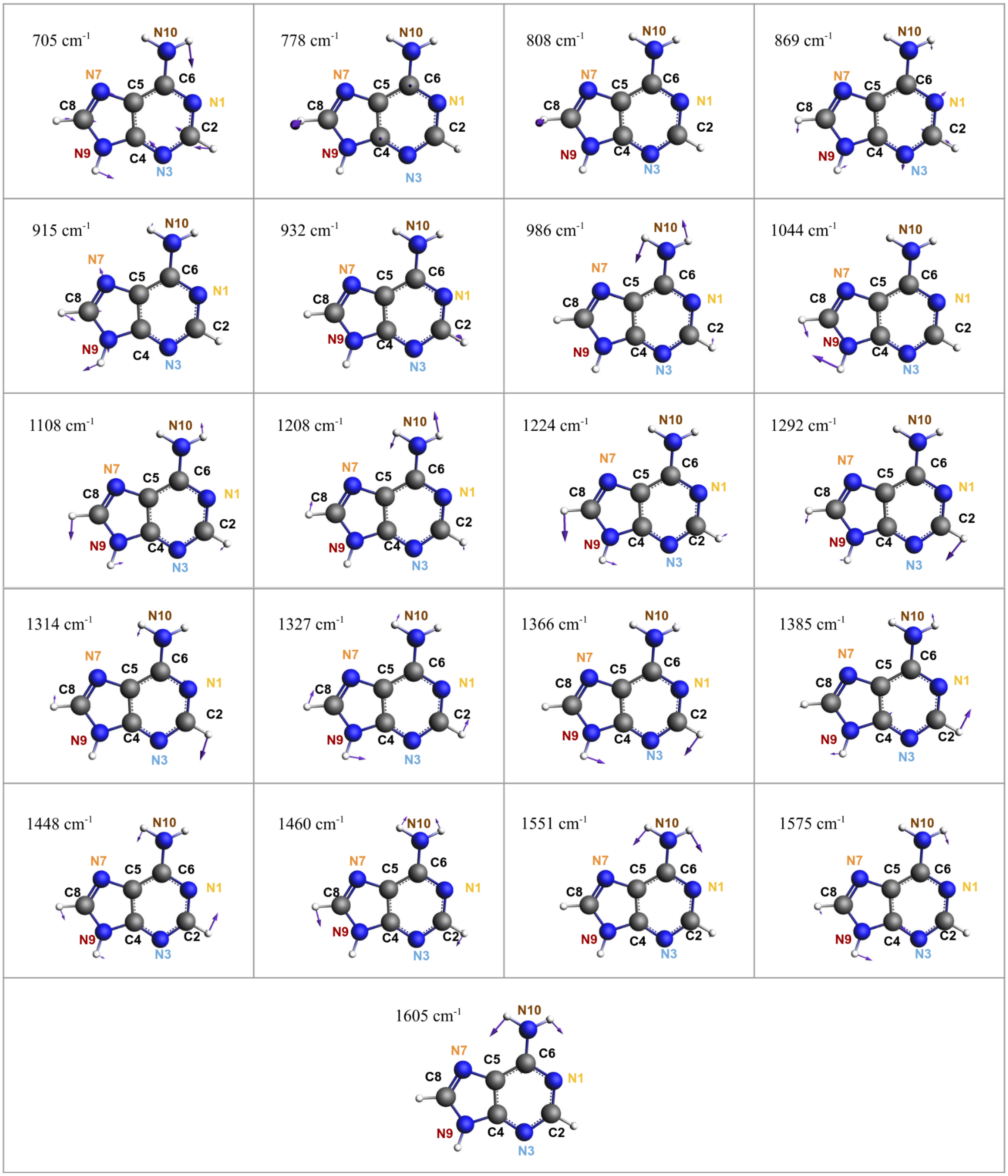

Figure S3: Vibrational normal modes of ADE in gas phase from 700 cm^{-1} to 1700 cm^{-1}.

Table S1: Average Enhancement Factor (AEF), Maximum Enhancement Factor (MEF) and normal mode associated to the MEF (i-MEF) for SEIRA (800-1700 cm^{-1}) and SERS (700-1700 cm^{-1}) spectra of ADE on Au_{10179} Ih. δ=bending/scissoring, ν=stretching, ρ=rocking, ω=wagging

SEIRA				
**CONFIG**	**AEF**	**MEF**	**i-MEF**	**Assignment**
v_N1	3.05	28.98	1550 cm^{-1}	**in-plane**; $\delta(NH_2)$, δ(C2-H), ν(C5-C6), ν(C4-C5)
f_N1	1.43	5.06	986 cm^{-1}	**in-plane**; $\rho(NH_2)$, δ(C2-H), ν(N1-C6)
v_N3	6.70	26.36	1550 cm^{-1}	**in-plane**; $\delta(NH_2)$, δ(C2-H), ν(C5-C6), ν(C4-C5)
f_N3	3.33	4.62	1448 cm^{-1}	**in-plane**; ν(N7-C8), ν(N1-C6), ν(C2-N3), δ(C2-H), δ(C8-H)
v_N7	3.48	41.62	1460 cm^{-1}	**in-plane**; $\delta(NH_2)$, ν(N7-C8), ν(C6-N10), δ(C2-H), δ(C8-H)
f_N7	2.15	4.82	1224 cm^{-1}	**in-plane**; δ(C8-H), δ(N9-H), δ(C2-H)
v_N9	5.14	30.55	1460 cm^{-1}	**in-plane**; $\delta(NH_2)$, ν(N7-C8), ν(C6-N10), δ(C2-H), δ(C8-H)
f_N9	3.49	5.12	1550 cm^{-1}	**in-plane**; $\delta(NH_2)$, δ(C2-H), ν(C5-C6), ν(C4-C5)
v_N10	9.24	13.05	1605 cm^{-1}	**in-plane**; $\delta(NH_2)$, ν(C5-C6), ν(C6-N10)
f_N10	3.68	4.62	1605 cm^{-1}	**in-plane**; $\delta(NH_2)$, ν(C5-C6), ν(C6-N10)
v_p	0.35	77.92	1460 cm^{-1}	**in-plane**; $\delta(NH_2)$, ν(N7-C8), ν(C6-N10), δ(C2-H), δ(C8-H)
f_p	0.09	5.95	932 cm^{-1}	**out-of-plane**; ω(C2-H)
**SERS**				
**CONFIG**	**AEF**	**MEF**	**i-MEF**	**Assignment**
v_N1	$2.34 \cdot 10^2$	$2.94 \cdot 10^3$	869 cm^{-1}	**in-plane**; def R6(sqz group N1-C2-N3), R5(ν(C5-N7))
f_N1	17.50	28.24	1460 cm^{-1}	**in-plane**; ν(N7-C8), δ(C8-H), δ(C2-H), $\delta(NH_2)$
v_N3	$2.53 \cdot 10^2$	$5.20 \cdot 10^3$	869 cm^{-1}	**in-plane**; def R6(sqz group N1-C2-N3), R5 (νC5-N7)
f_N3	10.38	30.78	1108 cm^{-1}	**in-plane**; δ(C8-H), δ(N10-H11), ν(C4-N9), ν(N3-C4), ν(C6-N10)
v_N7	$2.23 \cdot 10^2$	$2.35 \cdot 10^3$	915 cm^{-1}	**in-plane**; def R5 (sqz group N7-C8-N9)
f_N7	9.43	28.11	1108 cm^{-1}	**in-plane**; δ(C8-H), δ(N10-H11), ν(C4-N9), ν(N3-C4), ν(C6-N10)
v_N9	33.98	$3.47 \cdot 10^2$	1385 cm^{-1}	**in-plane**; ν(C4-N9), ν(C4-C5), ν(C6-N10), ν(N7-C8), δ(C2-H)
f_N9	6.29	26.87	915 cm^{-1}	**in-plane**; def R5 (sqz group N7-C8-N9)
v_N10	99.83	$1.30 \cdot 10^3$	1385 cm^{-1}	**in-plane**; ν(C4-N9), ν(C4-C5), ν(C6-N10), ν(N7-C8), δ(C2-H)
f_N10	7.34	26.00	915 cm^{-1}	**in-plane**; def R5 (sqz group N7-C8-N9)
v_p	47.62	$6.35 \cdot 10^3$	778 cm^{-1}	**out-of-plane**; ω(C8-H), def R6 (ω(C4-C5-C6))
f_p	0.46	14.88	1385 cm^{-1}	**in-plane**; ν(C4-N9), ν(C4-C5), ν(C6-N10), ν(N7-C8), δ(C2-H)

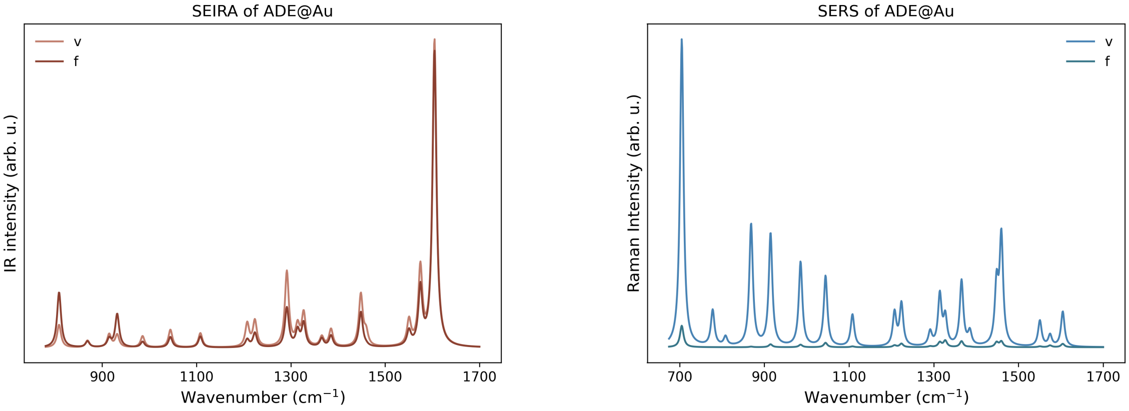

Figure S4: QM/ωFQFμ SEIRA (left) and SERS (right) spectra of ADE calculated by averaging the 6 configurations on the vertex (v) and face (f) of Au_{10179} Ih.

a)

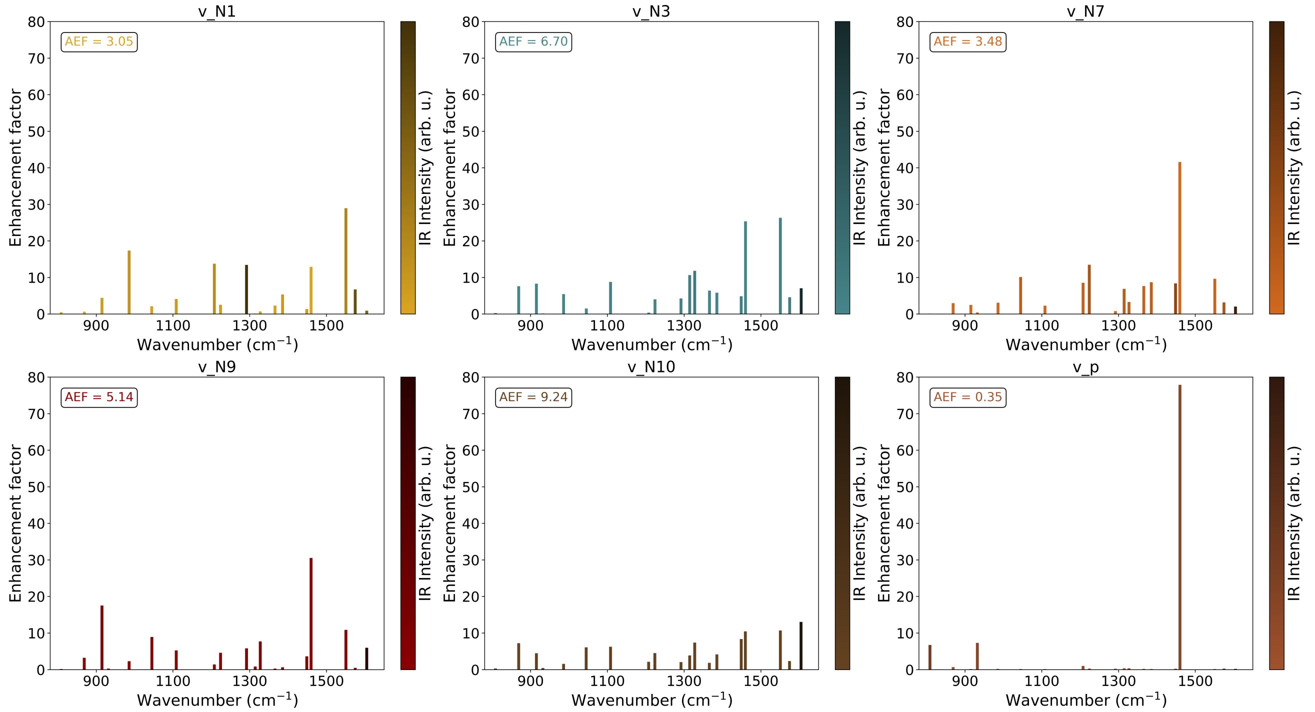

b)

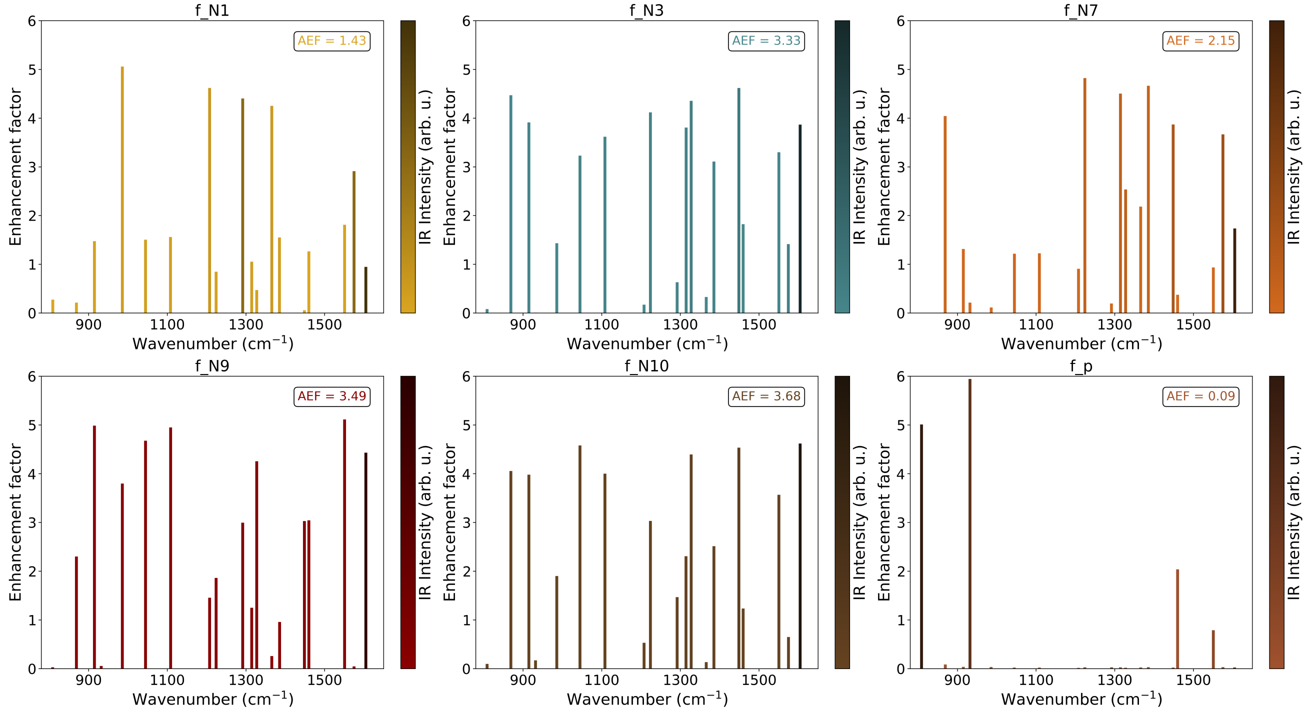

Figure S5: SEIRA enhancement factors computed for each normal mode of ADE adsorbed on Au NP (vertex, a; face, b). EFs are plotted with a palette following SEIRA intensities. AEF values are also reported.

a)

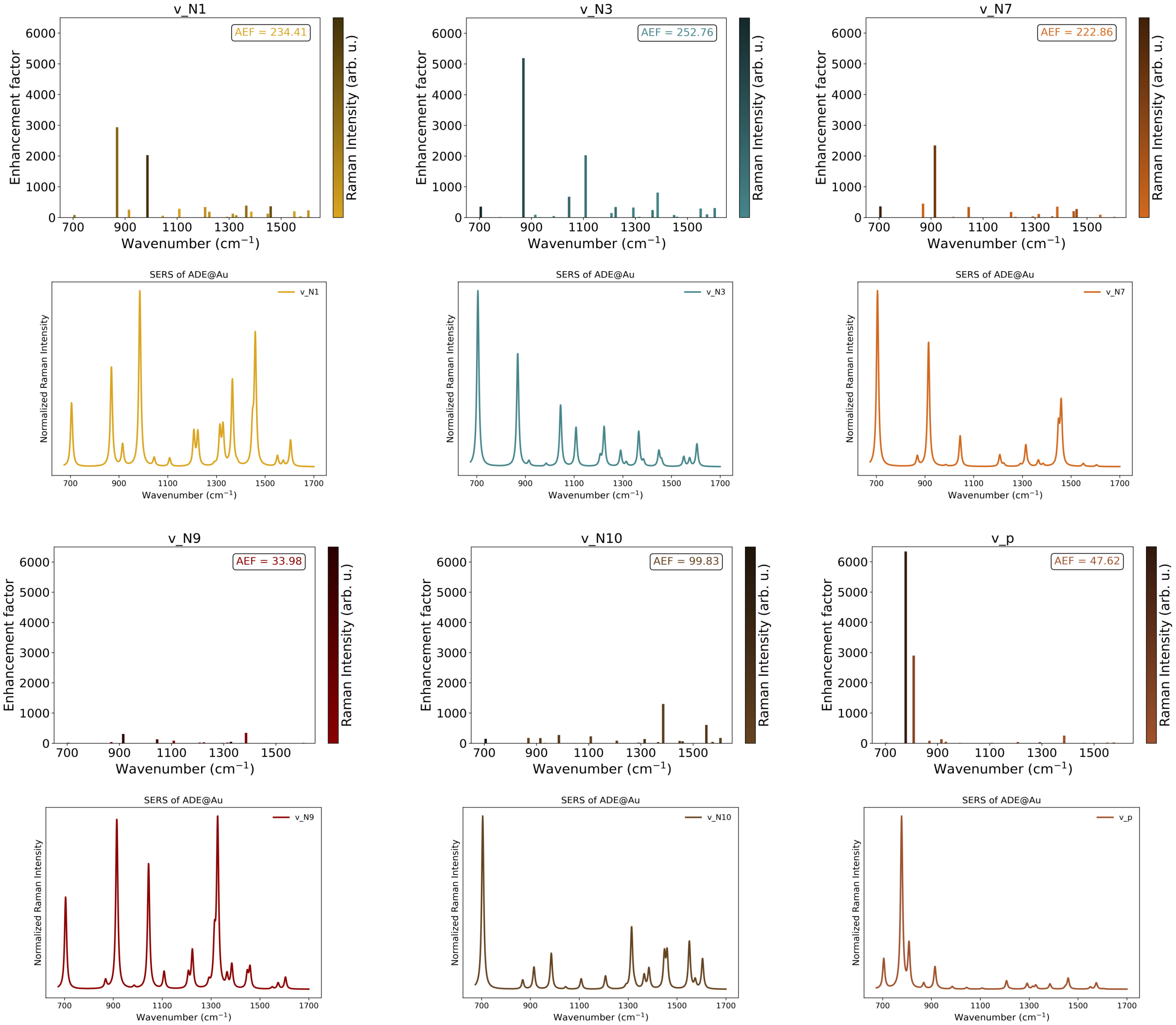
v_N1
AEF = 234.41
Enhancement factor
Raman Intensity (arb. u.)
Wavenumber (cm^{-1})
v_N3
AEF = 252.76
v_N7
AEF = 222.86
SERS of ADE@Au
Normalized Raman Intensity
v_N9
AEF = 33.98
v_N10
AEF = 99.83
v_p
AEF = 47.62

b)

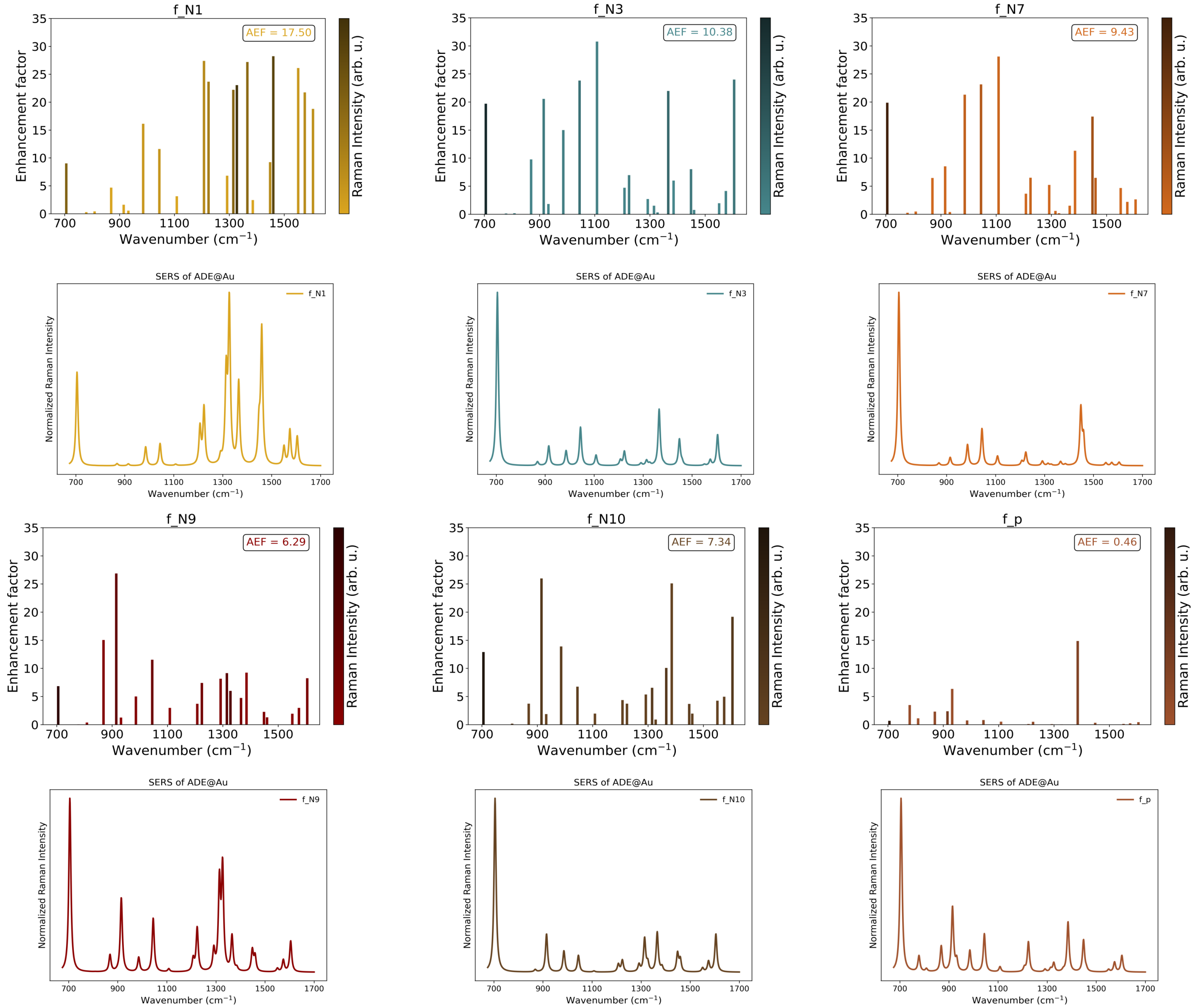

Figure S5: Normalized SERS spectra and enhancement factors (EFs) computed for each normal mode of the various configurations of ADE adsorbed on Au NP (vertex, a; face, b).

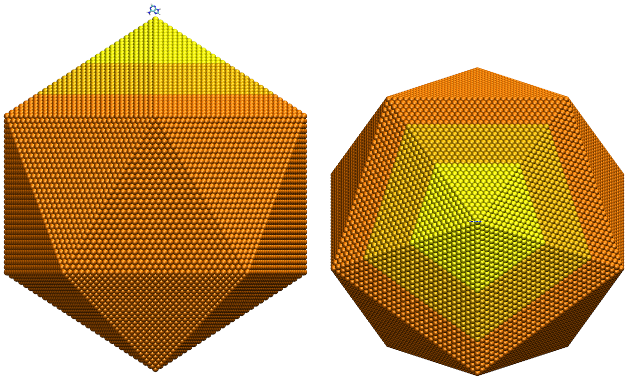

Figure S6: ADE adsorbed on the vertex of three Au NPs of increasing size: Au_{10179} (yellow), Au_{49049} (gold), and Au_{104223} (orange) nanoparticles in v_N7 configuration.

a)

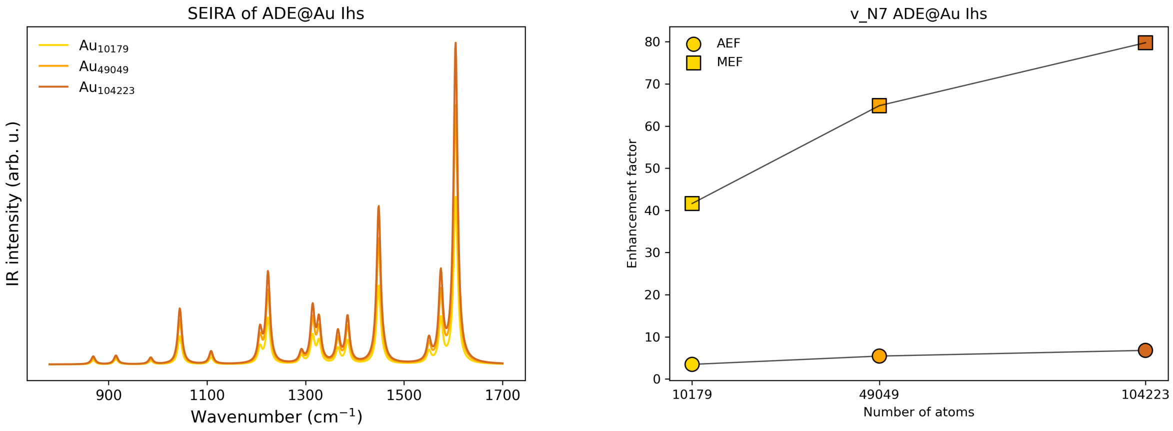

b)

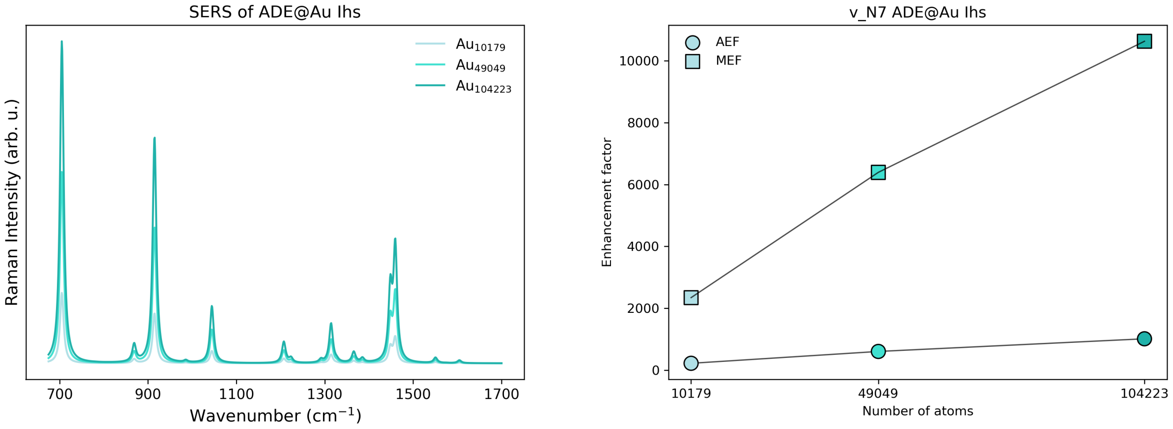

Figure S7: QM/ωFQFμ SEIRA (a) and SERS (b) spectra of ADE adsorbed on the vertex of three Au NPs of increasing size in v_N7 configuration (left). Corresponding AEF and MEF as a function of the NP number of atoms (right). The i-MEF is the normal mode at 1460 cm^{-1} for SEIRA and at 915 cm^{-1} for SERS.

Table S2: Radius (Å), Number of Atoms and PRF values for each graphene disk (GD) computed at the ωFQ level.

**System**	**Radius** (Å)	**Number of Atoms**	**PRF** (cm^{-1})
GD24	120	17269	2258
GD28	140	23485	2097
GD32	160	30724	1936
GD36	180	38893	1815
GD40	200	47998	1734
GD50	250	75178	1573
GD60	300	108236	1412
GD70	350	147331	1331
GD80	400	192432	1250
GD90	450	243567	1170
GD100	500	300695	1089

Table S3: ωFQ GD32 PRF values as a function of the Fermi energy (E_F).

E_F (eV)	**PRF** (cm^{-1})
0.40	1936
0.28	1629
0.23	1468
0.20	1371
0.17	1258
0.13	1081
0.11	1016
0.09	919

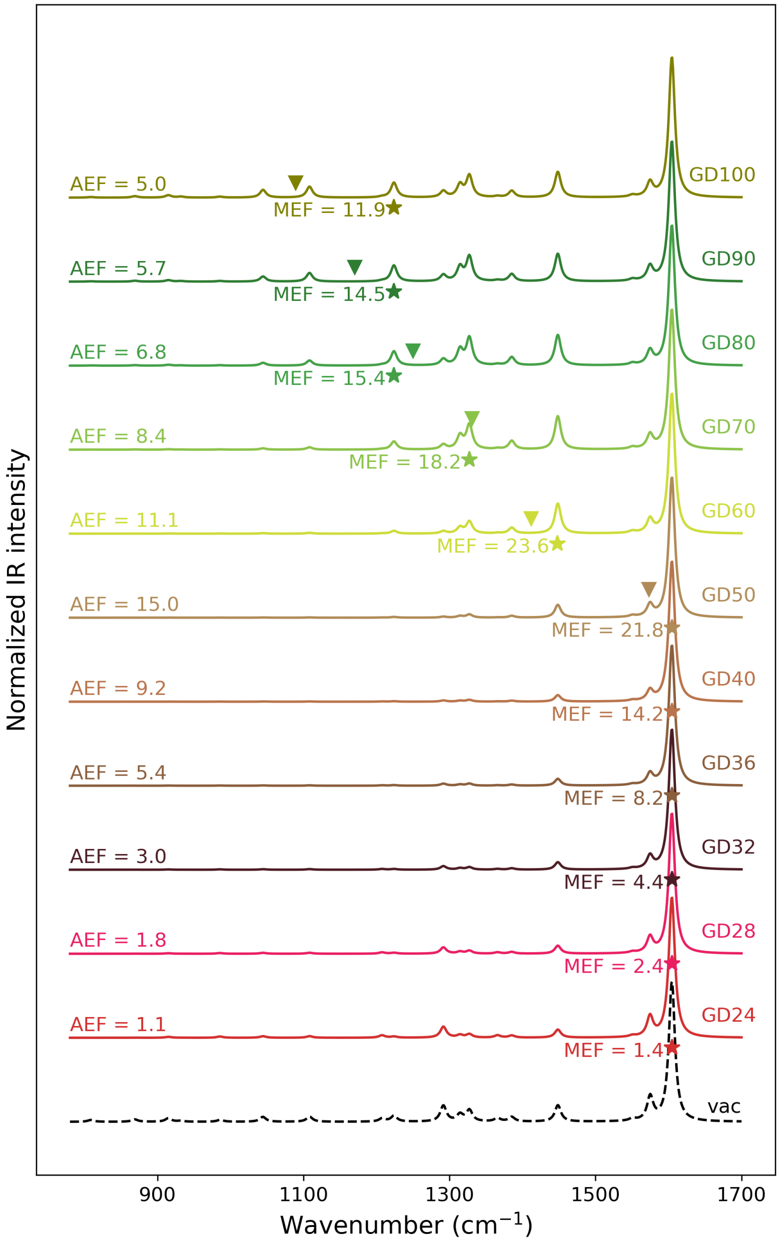

Figure S8: Normalized QM/ωFQ SEIRA spectra of ADE in the N1 configuration adsorbed on GDs of increasing size. Stars denote the i-MEFs; triangles indicate the PRFs. AEF and MEF values are also reported.

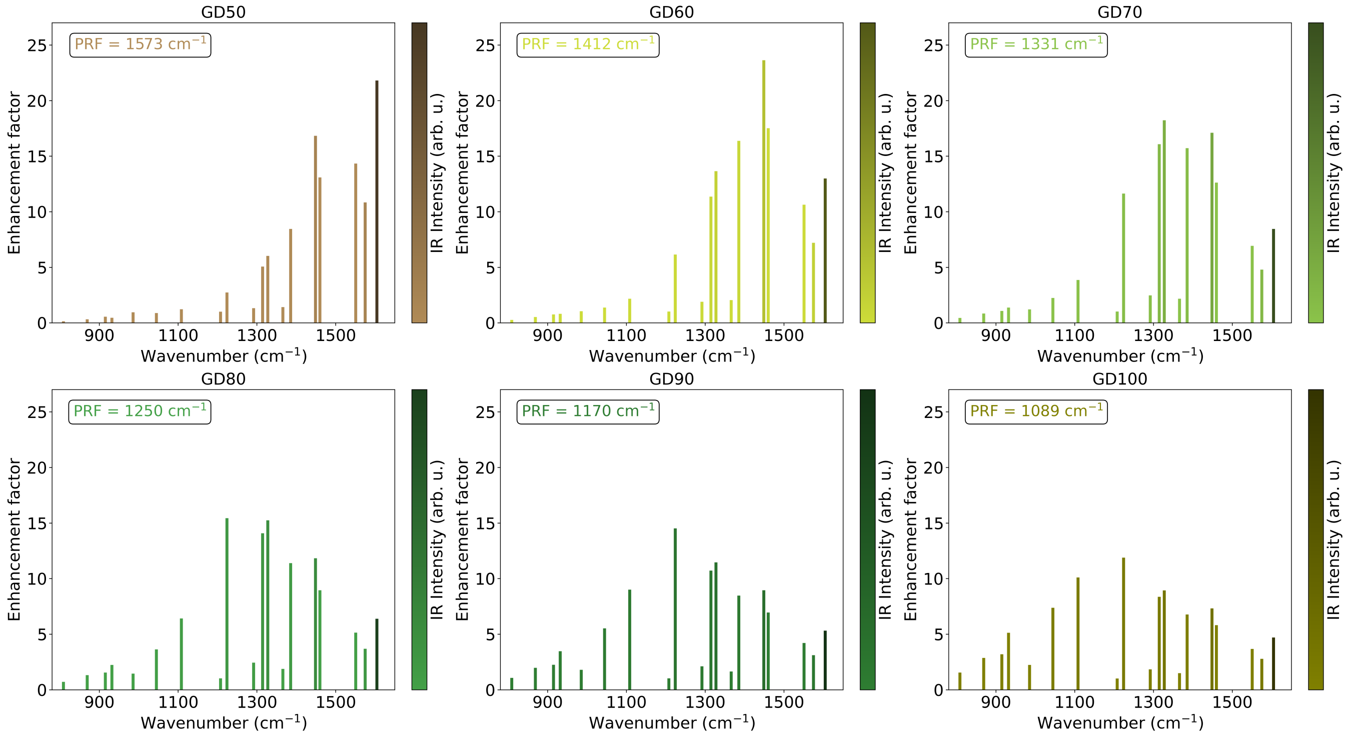

Figure S9: SEIRA enhancement factors computed for each normal mode of ADE in the N1 configuration on GDs of increasing size. EFs are plotted with a palette following SEIRA intensities.

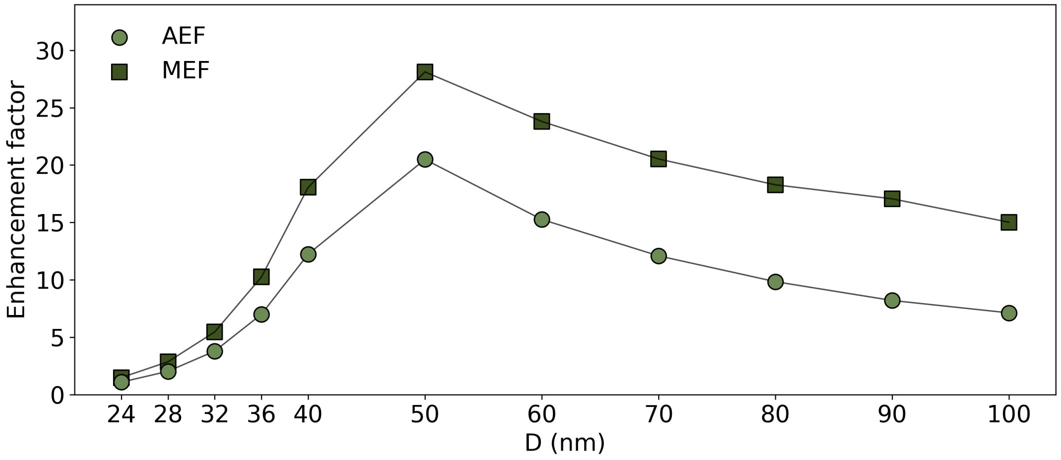

Figure S10: SEIRA AEF and MEF values calculated for ADE in the parallel configuration on GDs of increasing size.

Table S4: Average Enhancement Factors (AEF), Maximum Enhancement Factors (MEF), and normal mode associated with the MEF (i-MEF) of the SEIRA spectra of ADE in the parallel configuration adsorbed on GDs of increasing size (the corresponding PRFs are also listed). δ=bending/scissoring, ν=stretching, ρ=rocking

DISK	PRF	AEF	MEF	i-MEF	Assignment
GD24	2258 cm^{-1}	1.08	1.48	1605 cm^{-1}	**in-plane**; $\delta(NH_2)$, ν(C5-C6), ν(C6-N10)
GD28	2097 cm^{-1}	2.03	2.86	1605 cm^{-1}	**in-plane**; $\delta(NH_2)$, ν(C5-C6), ν(C6-N10)
GD32	1936 cm^{-1}	3.79	5.46	1605 cm^{-1}	**in-plane**; $\delta(NH_2)$, ν(C5-C6), ν(C6-N10)
GD36	1815 cm^{-1}	7.00	10.25	1605 cm^{-1}	**in-plane**; $\delta(NH_2)$, ν(C5-C6), ν(C6-N10)
GD40	1734 cm^{-1}	12.24	18.06	1605 cm^{-1}	**in-plane**; $\delta(NH_2)$, ν(C5-C6), ν(C6-N10)
GD50	1573 cm^{-1}	20.49	28.12	1575 cm^{-1}	**in-plane**; δ(N9-H), δ(C8-H), ν(N3-C4), ν(N1-C6), ν(C5-N7), ν(N7-C8)
GD60	1412 cm^{-1}	15.26	23.80	1448 cm^{-1}	**in-plane**; ν(N7-C8), ν(N1-C6), ν(C2-N3), δ(C2-H), δ(C8-H)
GD70	1331 cm^{-1}	12.09	20.53	1327 cm^{-1}	**in-plane**; ν(C8–N9), ν(C6–N1), ν(N3–C4), δ(C8–H), δ(N9–H)
GD80	1250 cm^{-1}	9.84	18.28	1292 cm^{-1}	**in-plane**; ν(C2-N3), ν(C5-N7), δ(C2-H), δ(C8-H), δ(N9-H)
GD90	1170 cm^{-1}	8.20	17.06	1208 cm^{-1}	**in-plane**; $\rho(NH_2)$, δ(C8-H), ν(C5-N7), ν(N1-C2)
GD100	1089 cm^{-1}	7.12	15.02	1108 cm^{-1}	**in-plane**; δ(C8-H), δ(N10-H), ν(C4-N9), ν(N3-C4), ν(C6-N10)

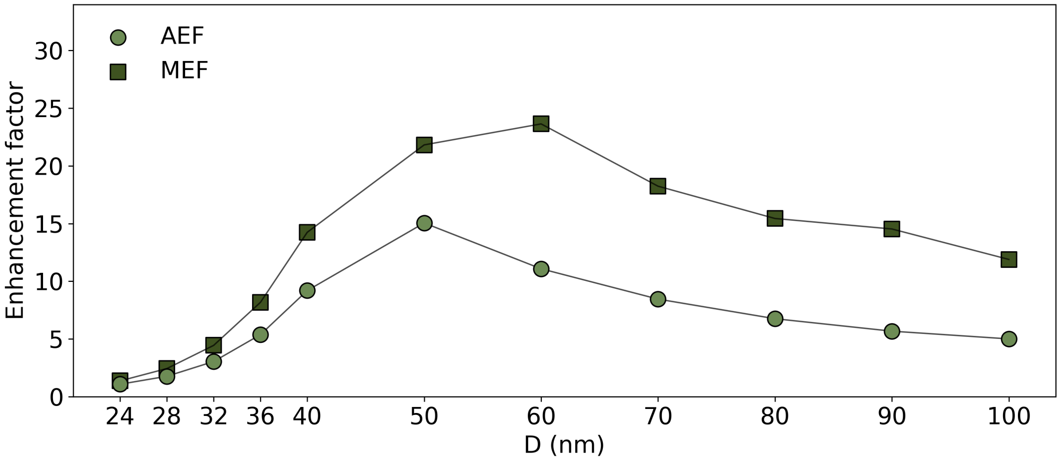

Figure S11: SEIRA AEF and MEF values calculated for ADE in the N1 configuration on GDs of increasing size.

Table S5: Average Enhancement Factors (AEF), Maximum Enhancement Factors (MEF), and normal mode associated with the MEF (i-MEF) of the SEIRA spectra of ADE in the parallel configuration adsorbed on GD32 as a function of the Fermi energy (the corresponding PRFs are also listed). δ=bending/scissoring, ν=stretching, ρ=rocking, ω=wagging

E_F	PRF	AEF	MEF	i-MEF	Assignment
0.28 eV	1629 cm^{-1}	19.45	27.98	1605 cm^{-1}	**in-plane**; $\delta(NH_2)$, ν(C5-C6), ν(C6-N10)
0.23 eV	1468 cm^{-1}	16.95	23.01	1448 cm^{-1}	**in-plane**; ν(N7-C8), ν(N1-C6), ν(C2-N3), δ(C2-H), δ(C8-H)
0.20 eV	1371 cm^{-1}	13.05	21.70	1385 cm^{-1}	**in-plane**; ν(C4-N9), ν(C4-C5), ν(C6-N10), ν(N7-C8), δ(C2-H)
0.17 eV	1258 cm^{-1}	9.91	18.23	1292 cm^{-1}	**in-plane**; ν(C2-N3), ν(C5-N7), δ(C2-H), δ(C8-H), δ(N9-H)
0.13 eV	1081 cm^{-1}	6.58	14.30	1108 cm^{-1}	**in-plane**; δ(C8-H), δ(N10-H11), ν(C4-N9), ν(N3-C4), ν(C6-N10)
0.11 eV	1016 cm^{-1}	5.71	12.69	1044 cm^{-1}	**in-plane**; δ(N9-H), δ(C8-H), ν(C8-N9)
0.09 eV	919 cm^{-1}	5.09	10.31	915 cm^{-1}	**in-plane**; def R5 (sqz group N7-C8-N9)

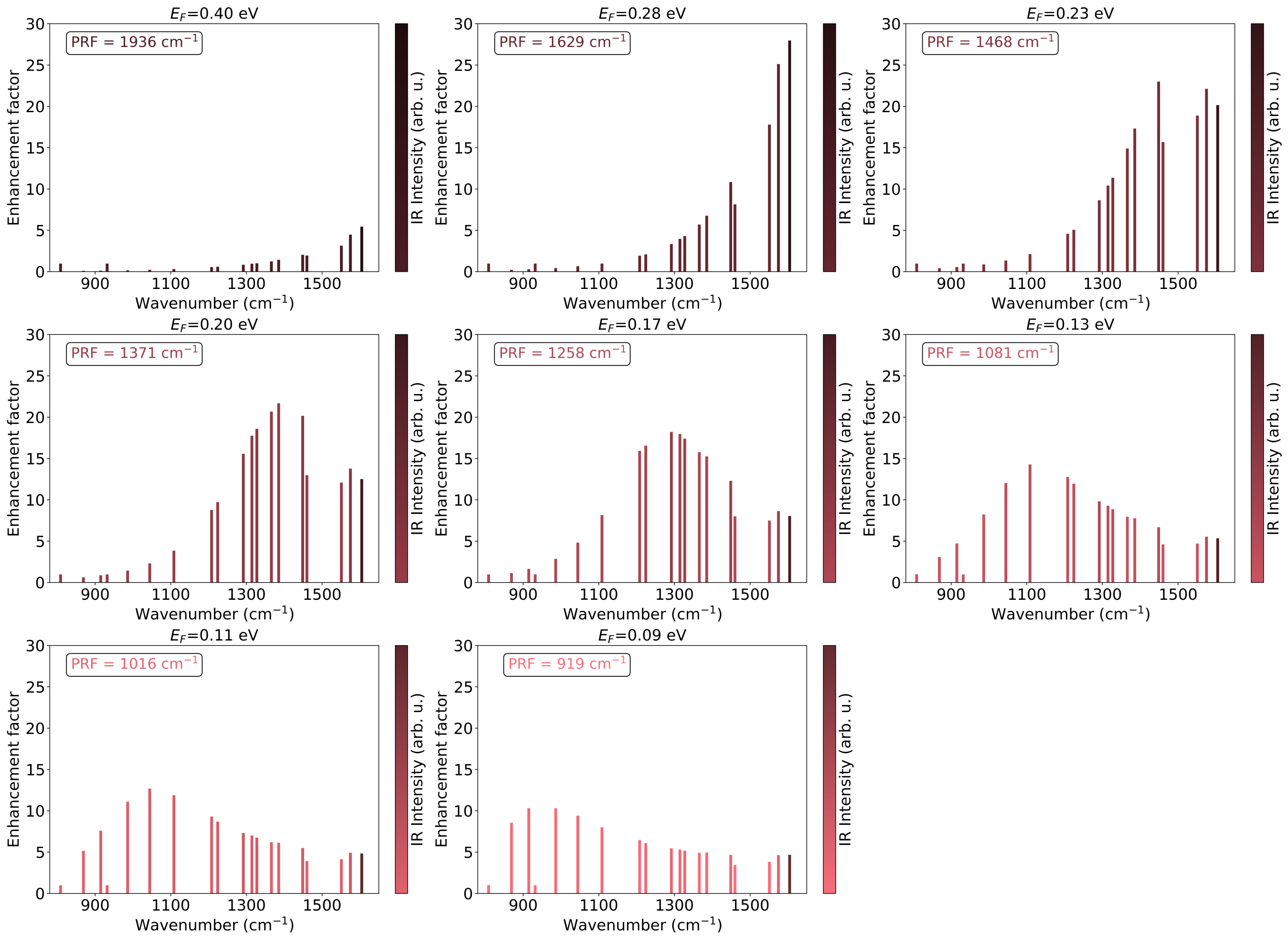

Figure S12: SEIRA enhancement factors (EFs) computed for each normal mode of ADE in the parallel configuration adsorbed on GD32 as a function of the Fermi energy. EFs are plotted with a palette following SEIRA intensities. PRF values are also reported.
